# Synchronous osteoclastoma and anaplastic astrocytoma: A case report

**DOI:** 10.3892/ol.2013.1567

**Published:** 2013-09-06

**Authors:** BINHUA LI, BIN ZHANG, WEIQI REN, XIANG XIAO, MIN DAI

**Affiliations:** 1 Department of Orthopaedics, The First Affiliated Hospital of Nanchang University Medical School, Nanchang, Jiangxi 330006, P.R. China; 2 Department of Pathology, The First Affiliated Hospital of Nanchang University Medical School, Nanchang, Jiangxi 330006, P.R. China

**Keywords:** multiple primary neoplasms, osteoclastoma, astrocytoma

## Abstract

Multiple primary neoplasms are defined as multiple occurrences of malignant neoplasms of differing histological origin in the same individual. The present study describes the case of a 46-year-old male who suffered from two synchronous primary malignant neoplasms, an osteoclastoma of the left femoral trochanter and an anaplastic astrocytoma of the the Sylvian fissure area in the brain. At the 6-month follow-up, the patient presented no problems following the aggressive treatment, including surgical resection, radiation therapy and chemotherapy. To the best of our knowledge, this is the first study in the medical literature of such a presentation.

## Introduction

Multiple primary neoplasms are defined as cases of two or more simultaneous abnormal growths of tissue, which are presumed to be of separate origin in one person. The neoplasms may be histologically similar or different and may be identified in the same or different sites (1). With the improvement of diagnostic techniques, the progressive lengthening of life expectancy and the increased long-term survival of patients with malignancy, there have recently been an subsequently increasing number of patients diagnosed with multiple primary neoplasms.

Osteoclastoma and anaplastic astrocytoma are relatively common malignant neoplasms in their respective fields. Synchronous osteoclastoma and anaplastic astrocytoma in the same individual, however, has not been previously reported. The present study describes the case of a 46-year-old male patient with synchronous primary malignant tumors of the femoral trochanter and brain.

## Case report

**Figure 1:**
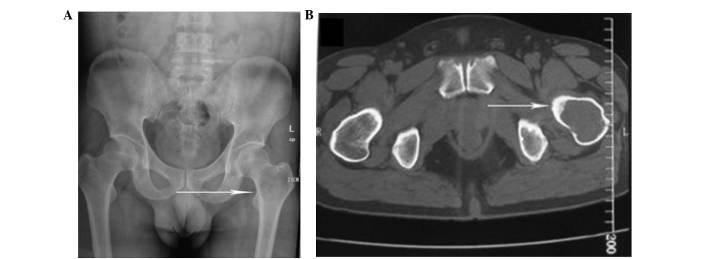
(A) Radiograph and (B) computed tomography (CT) images of the pelvis showing a low-signal intensity in the left femoral trochanter (white arrow).

**Figure 2:**
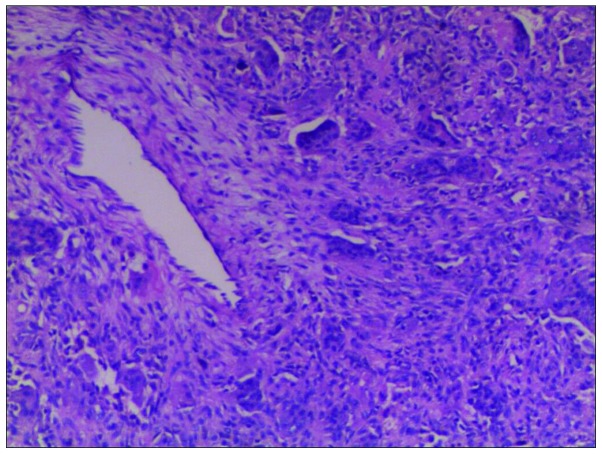
A post-operative pathological examination showing typical histology with multinuclear giant cells between numerous histocytes, without obvious nuclear atypia. (HE staining; magnification, ×200).

**Figure 3:**
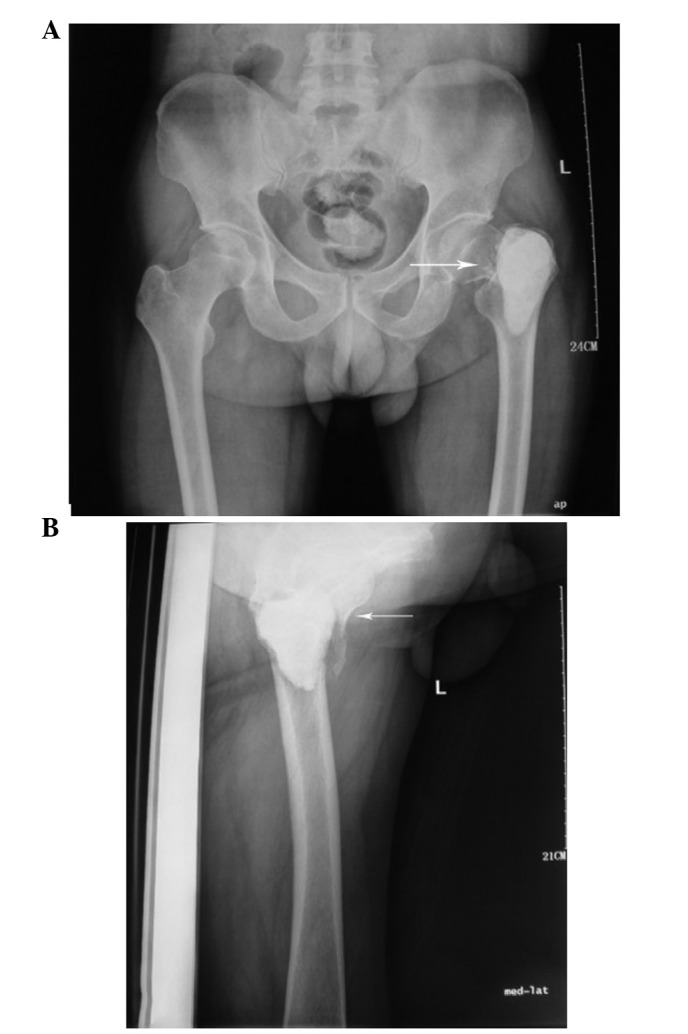
(A) Frontal and (B) lateral x-ray images of the pelvis showing a fracture of the left femoral neck (white arrow).

**Figure 4:**
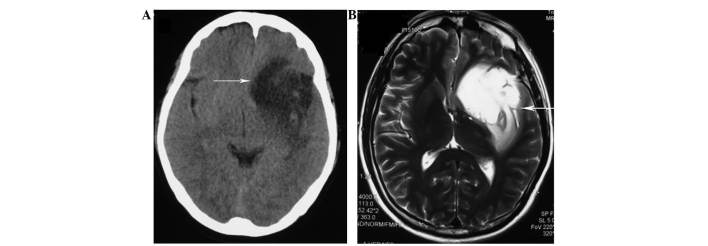
Cranial (A) computed tomography (CT) and (B) magnetic resonance imaging (MRI) showing an altered signal intensity in the left frontotemporal region of the brain (white arrow).

**Figure 5:**
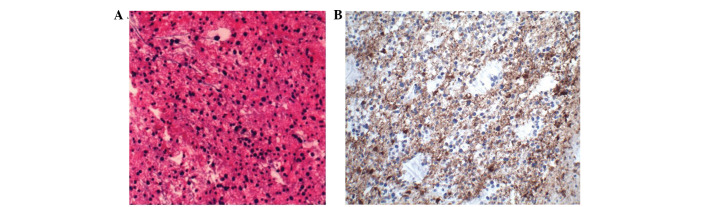
(A) HE staining showing bigger, irregular and hyperchromatic nuclei of the tumor cells and the incarnadined cytoplasms with unclear boundaries (magnification, ×200). (B) Immunohistochemical staining showing the tumor cells expressing glial fibrillary acidic protein (GFAP; magnification, ×200).

**Figure 6:**
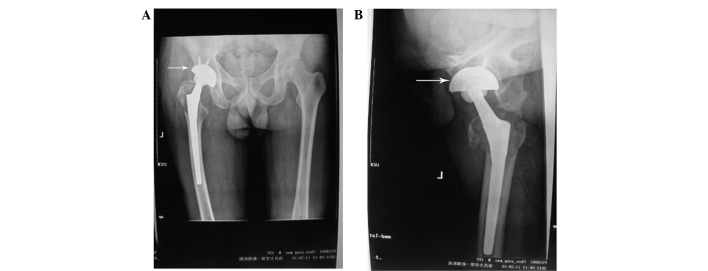
Post-operative (A) frontal and (B) lateral radiograph following the total hip arthroplasty (THA) of the left hip showing that the position of the the artificial joint prosthesis is stable (white arrow).

A 46-year-old male presented to the Department of Orthopedics, The First Affiliated Hospital of Nanchang University Medical School (Nanchang, Jiangxi, China) with a complaint of intermittent pain in the left hip for six months and aggravation of this symptom one month previous to admittance. The medical and family history of the patient was not significant. The general physical examination was normal, with the exception of limited movement and percussion pain in the left hip. A central nervous system examination revealed normal mental capabilities, a functioning motor and sensory system and no neck rigidity. All the cranial nerves were intact. X-ray and computed tomography (CT; Fig. 1) of the left hip revealed a femoral trochanteric lesion and a suspected osteoclastoma. The patient underwent left femoral tumor curettement with bone cement implantation. Intraoperative and post-operative histological examinations revealed an osteoclastoma (Fig. 2). One week after the surgery, the patient was discharged without any complications. However, the patient was admitted to hospital again two weeks later due to a sudden epileptic seizure that lasted for 4–5 min. The patient also felt significant pain in the left hip subsequent to regaining consciousness. X-ray of the pelvis (Fig. 3) revealed a fracture of the left femoral neck and CT of the brain (Fig. 4A) revealed a lamellar and low-density shadow in the left frontotemporal region of the brain. Cranial magnetic resonance imaging (MRI; Fig. 4B) revealed an abnormal signal intensity in the left frontotemporal region of the brain, indicating a suspected metastatic tumor or glioma. The patient underwent a left lateral fissure craniotomy and a gross total resection of the lesion. An anaplastic astrocytoma (World Health Organization scale grade III) was diagnosed following an examination of a tissue sample (Fig. 5). Subsequently, the patient underwent an additional total hip arthroplasty (THA) due to the fracture of the left femoral neck (Fig. 6). The post-operative period was uneventful and the patient was referred to the Department of Oncology two weeks later. The patient was administered adjuvant radiotherapy with (total dose, 60 Gy) and 6 cycles of chemotherapy as follows: Temozolomide was administered at a dose of 250 mg on days 1–5 and the course was repeated every 28 days. The patient was doing well with no evidence of local or distant recurrence more than six months after the surgery. Approval for this study was obtained from the ethical review committee of The First Affiliated Hospital of Nanchang University Medical School and all the investigations were conducted in conformity with the ethical principles of research. Informed consent was obtained from the patient for participation in the study.

## Discussion

Multiple primary malignant tumors in an individual are relatively rare. In a review of the literature on multiple primary malignant neoplasms, the overall incidence of multiple primary malignancies has been recorded as 0.73–11.7% (2).

In 1889, Billroth (3) published the first documented occurrence of multiple primary malignancies. In 1932, Warren and Gates (4) recommended the following standard for the classification of multiple primary neoplasms: i) Each tumor must be distinct; ii) each tumor must present a definite picture of malignancy; and iii) the chance of one tumor being a metastasis of the other must be excluded. In 1977, Moertel (5) proposed the following definitions of multiple primary neoplasms, which are classifications that remain widely used today: i) Multifocal, the two distinct malignancies arise in the same organ or tissue; ii) systematic, arising in anatomically or functionally allied organs of the same system; iii) paired, arising in paired organs; and iv) random, occurring as a co-incidental or accidental association in unrelated sites. Moertel *et al*(6) also classified multiple primary neoplasms into two groups, synchronous, where the neoplasms appear at the same time or within 6 months, and metachronous, where the malignancies develop by turn (>6 months). Howe (1) classified multiple primary neoplasms as those that are observed at the same time or within two months as synchronous multiple primary neoplasms and these cancers develop at more than a two-month interval as metachronous multiple primary neoplasms.

The pathogenesis of multiple primary neoplasms remains unclear, but previous studies (7,8) have indicated that certain mechanisms, including genetic and immunological susceptibility, family history, perennial exposure to carcinogens, radiotherapy and chemotherapy for primary neoplasms, may play significant roles in the occurrence of multiple primary neoplasms. Additionally, Schottenfeld (9) believed that the two neoplasms may possess a shared etiology if the incidence of a subsequent neoplasm is significantly higher among those with a previous primary cancer.

In the patient of the present study, the malignant features were histopathologically proven in each tumor. Each tumor was pathologically categorized as a separate type. The tumor that was detected in the left femoral trochanter was an osteoclastoma and the tumor in the brain was an anaplastic astrocytoma. These findings may also support the fact that these two neoplasms occurred in a random and synchronous manner.

Osteoclastoma is one of the most obscure and discussed bone tumors, and the histogenesis of the tumor is uncertain as the histology does not predict the clinical outcome. The World Health Organization has classified osteoclastoma as ‘an aggressive, potentially malignant lesion’ (10). Previous studies have shown that 80% of osteoclastomas have a benign course, with a local rate of recurrence of 20–50%, and that ~10% undergo malignant transformation at recurrence, with 1–4% experiencing pulmonary metastases even in benign cases (11–14).

Astrocytoma is the most common neuroglial tumor, but is more commonly located in the cerebrum, leptomeninges and spinal cord, and is usually present with other associated focal neurological signs and symptoms (15). The designation of a tumor as an anaplastic astrocytoma reflects a distinct histological classification of malignant glioma characterized by an abundance of pleomorphic astrocytes with evidence of mitosis. Although there has been advancement in available treatments, including surgical resection, radiation therapy and chemotherapy, the majority of tumors recur within a few years and these recurrent tumors are more refractory to subsequent therapies. The survival of patients with malignant glioma remains poor, with a median survival of 2 years for patients with anaplastic astrocytoma (16,17).

Proverbially, the early diagnosis of malignancy prior to the appearance of clinical symptoms is important, and screening procedures, including pathological and imaging examinations, should be emphasized as soon as possible. Furthermore, the possibility of a second or third malignant lesion should be considered for patients with primary cancer. The prognosis of patients with multiple primary malignant tumors may be determined independently by the stage of each malignancy. A study by Di Martino *et al*(18) identified that although the mean lifetime of 18 months and the 5-year survival rate of 11.9% have been observed in patients with synchronous neoplasms, an early diagnosis and aggressive treatment of surgery may prolong and improve the quality of the life of the patient.

The patient in the present study suffered from synchronous appearance of osteoclastoma and anaplastic astrocytoma, which is rare and previously unreported in the medical literature.
